# Hydrogen sulfide at the intersection of hypoxia and oxidative stress: implications for lung cancer progression and treatment – a narrative review

**DOI:** 10.1097/MS9.0000000000004731

**Published:** 2026-01-21

**Authors:** Emmanuel Ifeanyi Obeagu, Jomar L. Aban

**Affiliations:** a Department of Biomedical and Laboratory Science, Africa University, Mutare, Zimbabwe; b Department of Molecular Medicine and Haematology, Faculty of Health Sciences, Public Health, University of the Witwatersrand, Johannesburg, South Africa; c Don Mariano Marcos Memorial State University, Philippines

**Keywords:** hydrogen sulfide, hypoxia, lung cancer, oxidative stress, tumor microenvironment

## Abstract

Lung cancer remains a leading cause of cancer-related mortality worldwide, with a pathogenesis deeply influenced by the tumor microenvironment. Two central and interrelated factors –hypoxia and oxidative stress – contribute significantly to tumor progression, angiogenesis, metabolic reprogramming, and therapeutic resistance. In recent years, hydrogen sulfide (H_2_S), traditionally viewed as a toxic gas, has gained recognition as a critical gasotransmitter with a regulatory role in both hypoxic and redox signaling pathways in cancer biology. Endogenously produced by enzymes such as CBS, CSE, and 3-MST, H_2_S can promote or inhibit tumorigenesis depending on the context. In lung cancer, H_2_S has been shown to modulate hypoxia-inducible factor activity, support mitochondrial bioenergetics under low oxygen tension, and influence ROS dynamics, thereby maintaining redox balance that favors tumor cell survival. The complex crosstalk between H_2_S, hypoxia, and oxidative stress creates a permissive environment for tumor growth and immune evasion, but also offers potential vulnerabilities that can be therapeutically exploited. Targeting H_2_S signaling has emerged as a promising avenue in lung cancer management. Both inhibition and controlled supplementation of H_2_S are under investigation as strategies to disrupt tumor adaptation to hypoxia and oxidative stress. This review highlights the dualistic nature of H_2_S in lung cancer progression, explores its mechanisms of action in the context of hypoxic and oxidative stress pathways, and discusses the diagnostic and therapeutic potential of modulating the H_2_S axis for improved clinical outcomes.

## Introduction

Lung cancer remains one of the most formidable challenges in oncology, accounting for the highest number of cancer-related deaths globally. Despite advances in diagnostics and treatment modalities, the prognosis for patients with lung cancer remains poor, largely due to late-stage detection, rapid metastasis, and resistance to therapy. At the heart of these pathophysiological processes lies the complex and dynamic tumor microenvironment (TME), which orchestrates a range of biological responses that favor tumor survival and expansion. Two fundamental and interconnected components of this microenvironment – hypoxia and oxidative stress – play a central role in lung cancer development and progression^[1–3]^. Hypoxia, a hallmark of rapidly growing solid tumors, arises when oxygen demand exceeds supply due to abnormal vascular architecture and poor perfusion. This low-oxygen environment activates hypoxia-inducible factors (HIFs), particularly HIF-1α, which regulate the transcription of numerous genes involved in angiogenesis, glucose metabolism, cell proliferation, and survival. Hypoxia not only enables tumor cells to adapt to hostile conditions but also contributes to therapeutic resistance, particularly against radiotherapy and certain chemotherapeutic agents whose efficacy relies on adequate oxygenation^[4–6]^.

**HIGHLIGHTS**

- Hydrogen sulfide (H_2_S) modulates lung cancer progression through mitochondrial regulation, redox balance, and hypoxia adaptation.
- Crosstalk between H_2_S, hypoxia, and oxidative stress shapes the tumor microenvironment.
- Elevated CBS/CSE expression correlates with poor prognosis and therapy resistance in lung cancer.
- Targeting H_2_S biosynthesis offers novel therapeutic strategies against hypoxic, redox-adapted tumors.
- H_2_S-related biomarkers hold diagnostic and prognostic promise in precision lung cancer management.

Concurrently, oxidative stress – defined by an imbalance between reactive oxygen species (ROS) and the antioxidant defense systems – emerges as both a driver and a consequence of cancer progression. Moderate levels of ROS can promote cell proliferation, angiogenesis, and inflammation, while excessive oxidative stress can damage cellular components, leading to genomic instability and apoptosis. Cancer cells often operate within a narrow redox window, maintaining ROS levels that are high enough to support malignancy but below the threshold for cytotoxicity. This fine-tuned redox balance is tightly linked to the hypoxic environment of tumors^[7–9]^. Recent research has spotlighted hydrogen sulfide (H_2_S), a gaseous signaling molecule endogenously produced by the enzymes cystathionine β-synthase (CBS), cystathionine γ-lyase (CSE), and 3-mercaptopyruvate sulfurtransferase (3-MST). Once considered merely toxic, H_2_S is now recognized as a critical regulator of various physiological and pathological processes, including vasodilation, neuromodulation, inflammation, and cellular metabolism. Intriguingly, H_2_S has emerged as a key modulator of both hypoxic and redox responses in cancer cells, placing it at the intersection of two pivotal drivers of lung cancer biology^[10,11]^.

In the context of lung cancer, H_2_S has been shown to influence tumor behavior through multiple pathways. It can stabilize HIF-1α under normoxic conditions, effectively mimicking hypoxia and sustaining the hypoxia-driven gene expression program. Additionally, H_2_S modulates mitochondrial function and biogenesis, supporting energy production and cell survival under metabolic stress. As an antioxidant, it scavenges ROS and upregulates endogenous antioxidant enzymes, thereby fine-tuning the redox status of cancer cells. However, this same cytoprotective mechanism may confer resistance to oxidative damage induced by therapy^[12,13]^. The dual nature of H_2_S – acting as both a tumor promoter and a potential therapeutic agent – makes it a compelling focus for investigation. While elevated levels of H_2_S-producing enzymes have been correlated with poor prognosis and aggressive behavior in lung tumors, studies have also demonstrated that exogenous H_2_S donors or inhibitors can modulate cancer progression depending on dosage, timing, and the redox state of the tumor. These contradictory findings underscore the complexity of H_2_S biology and the need to contextualize its role within the broader framework of hypoxia and oxidative stress^[14,15]^. This review aims to explore the intricate interplay between H_2_S, hypoxia, and oxidative stress in lung cancer.

## Aim

This review aims to explore the multifaceted role of H_2_S in the pathophysiological mechanisms of hypoxia and oxidative stress in lung cancer. It discusses how H_2_S influences tumor progression, angiogenesis, inflammation, metabolic reprogramming, and resistance to therapy, while also evaluating its potential as a diagnostic biomarker and therapeutic target.

## Methods

This narrative review was conducted to synthesize current evidence on the role of H_2_S at the interface of hypoxia and oxidative stress in lung cancer progression and treatment responsiveness. Although narrative in design, the review followed a structured and transparent approach to ensure methodological rigor.

### Search strategy

A comprehensive literature search was performed across PubMed/MEDLINE, Scopus, Web of Science, and Google Scholar from their inception to November 2025. Search terms included combinations of: “hydrogen sulfide,” “H2S,” “lung cancer,” “non-small cell lung cancer,” “small cell lung cancer,” “hypoxia,” “HIF-1α,” “oxidative stress,” “redox signaling,” “cancer metabolism,” “tumor microenvironment,” and “therapeutic resistance.” Boolean operators (AND/OR) and Medical Subject Headings (MeSH) were used to refine the search.

### Eligibility criteria

Studies were included if they:

1. Investigated endogenous or exogenous H_2_S in the context of lung cancer biology;

2. Explored interactions between H_2_S, hypoxia, or oxidative stress pathways;

3. Reported mechanistic insights, preclinical experiments, clinical correlations, or therapeutic implications;

4. Were peer-reviewed full-text articles written in English.

Exclusion criteria were: conference abstracts without full texts; non-lung cancer studies unless mechanistically relevant; studies focused exclusively on environmental sulfide toxicity; editorials or commentaries without primary data.

### Study selection and data extraction

Titles and abstracts were screened independently, followed by full-text assessment for relevance. Discrepancies were resolved through discussion and consensus. For each eligible study, key information was extracted, including experimental model, H_2_S-related mechanisms, interactions with hypoxia or oxidative stress pathways, and implications for tumor progression or therapy.

### Quality considerations

As narrative reviews do not employ formal risk-of-bias scoring, emphasis was placed on incorporating findings from high-quality mechanistic studies, well-characterized *in vitro* and *in vivo* models, and clinical evidence where available. Priority was given to studies with clear methodological descriptions, reproducible experimental designs, and mechanistic depth.

### Synthesis approach

A thematic synthesis approach was employed. Extracted data were organized into conceptual domains – H_2_S biosynthesis and signaling in lung cancer, crosstalk with hypoxia-inducible pathways, modulation of oxidative stress responses, and therapeutic implications. Divergent findings were highlighted where relevant to reflect current uncertainties and research gaps.

### H_2_S: a gasotransmitter in lung cancer biology

H_2_S has historically been regarded as a toxic environmental pollutant; however, in the last two decades, it has emerged as a biologically relevant gasotransmitter alongside nitric oxide (NO) and carbon monoxide (CO). Endogenously synthesized in mammalian tissues, H_2_S exerts a broad spectrum of physiological functions, including vasodilation, neuromodulation, angiogenesis, cytoprotection, and regulation of inflammatory pathways. In the context of cancer, particularly lung cancer, H_2_S has gained increasing attention due to its dualistic roles in promoting or suppressing tumorigenesis depending on concentration, cellular context, and tumor microenvironmental cues^[9]^. H_2_S is primarily produced via the transsulfuration pathway by three key enzymes: CBS, CSE, and 3-MST. These enzymes are differentially expressed in tissues and may localize to cytosolic or mitochondrial compartments, suggesting spatially compartmentalized functions of H_2_S. In lung cancer, upregulation of CBS and CSE has been documented in both non-small cell lung cancer (NSCLC) and small cell lung cancer, where they contribute to enhanced proliferation, invasion, and metabolic flexibility. Moreover, mitochondrial 3-MST-derived H_2_S supports bioenergetic adaptation under hypoxic stress, which is particularly relevant in the poorly vascularized regions of lung tumors^[10]^.

Functionally, H_2_S modulates several hallmarks of cancer. It influences mitochondrial respiration by stimulating cytochrome c oxidase activity at low concentrations and inhibiting it at high levels, thereby fine-tuning ATP production and redox signaling. H_2_S also promotes angiogenesis by upregulating vascular endothelial growth factor (VEGF) and enhancing endothelial cell migration, which supports tumor expansion and nutrient delivery. Additionally, H_2_S exhibits anti-apoptotic effects by preserving mitochondrial integrity and activating prosurvival kinases such as PI3K/Akt and ERK1/2. These actions collectively contribute to the adaptability and resilience of lung cancer cells under stress conditions like hypoxia and oxidative stress^[11,12]^. However, the role of H_2_S in lung cancer is not unequivocally pro-tumorigenic. Under certain conditions, particularly when produced in excess or delivered exogenously at high concentrations, H_2_S can induce cytotoxicity, disrupt mitochondrial function, and trigger oxidative damage. This paradox has prompted investigations into both H_2_S donors and inhibitors as potential therapeutic agents. For example, pharmacological inhibition of CBS or CSE has been shown to reduce tumor growth, impair angiogenesis, and sensitize cancer cells to chemotherapeutics and radiation. Conversely, H_2_S-releasing compounds have demonstrated selective toxicity against cancer cells in redox-stressed environments, highlighting its potential in redox-based therapy^[13]^. Importantly, the effects of H_2_S are highly context-dependent and influenced by the local microenvironment, including oxygen availability, pH, and interaction with other gasotransmitters. In lung cancer, which is characterized by heterogeneity in oxygenation and redox status, H_2_S signaling becomes intricately entwined with hypoxia-induced pathways and ROS dynamics. Understanding these interactions is essential for developing strategies that harness or inhibit H_2_S signaling in a clinically meaningful way^[14]^.

### Hypoxia and the lung tumor microenvironment

Hypoxia, defined as a deficiency in tissue oxygen levels, is a hallmark of the TME in solid malignancies such as lung cancer. Rapidly proliferating tumor cells often outpace their blood supply, resulting in regions of chronic and acute hypoxia within the tumor mass. In the lung, where oxygen exchange is a fundamental physiological process, the presence of hypoxia within tumor tissues is paradoxical and profoundly influences cancer progression, therapeutic response, and immune evasion. Hypoxia is not merely a consequence of tumor growth – it actively shapes the biology of cancer cells and the surrounding stroma through complex signaling networks^[15]^. The primary molecular mediator of hypoxic responses is the HIF family, particularly HIF-1α and HIF-2α. Under normoxic conditions, HIF-α subunits are hydroxylated by prolyl hydroxylases (PHDs), which target them for proteasomal degradation. Hypoxia inhibits this hydroxylation, leading to HIF-α stabilization, dimerization with HIF-β, and translocation to the nucleus. Once activated, HIFs upregulate a wide array of genes involved in angiogenesis (e.g., VEGF), glycolysis (e.g., GLUT1, LDHA), survival (e.g., BNIP3), and metastasis (e.g., CXCR4), all of which contribute to the aggressive phenotype of lung tumors^[16]^.

In lung cancer, hypoxia is a driving force behind metabolic reprogramming, also known as the Warburg effect, wherein cancer cells shift from oxidative phosphorylation to glycolysis even in the presence of oxygen. This metabolic shift not only supports rapid energy production but also limits mitochondrial ROS generation, aiding in survival under oxidative stress. Furthermore, hypoxia-induced changes in the extracellular matrix (ECM), such as increased stiffness and altered integrin signaling, promote epithelial–mesenchymal transition (EMT) and facilitate local invasion and distant metastasis, particularly to oxygen-poor niches like the bone marrow and brain^[17]^. The hypoxic TME also exerts profound immunosuppressive effects. It recruits and polarizes tumor-associated macrophages (TAMs) toward an M2-like phenotype, enhances regulatory T cell (Treg) function, and reduces cytotoxic T lymphocyte (CTL) activity. In addition, hypoxia upregulates immune checkpoint molecules such as PD-L1, contributing to immune escape and resistance to immunotherapy. This immunosuppressive landscape is particularly significant in lung cancer, where immune checkpoint inhibitors are a mainstay of treatment, yet their efficacy is often compromised by underlying hypoxia-driven mechanisms^[18,19]^.

Hypoxia further complicates therapeutic outcomes by promoting resistance to radiotherapy and chemotherapy. Radiation efficacy relies on oxygen to generate DNA-damaging free radicals; thus, hypoxic tumor regions are less susceptible to radiation-induced cell death. Hypoxia also induces the expression of drug efflux transporters (e.g., MDR1), anti-apoptotic proteins (e.g., Bcl-2), and autophagy-related genes, all of which contribute to chemotherapy resistance. These adaptations create a sanctuary for tumor cells, allowing them to survive and repopulate after treatment^[20]^. The spatial and temporal heterogeneity of hypoxia in lung tumors poses a major challenge for effective treatment. Tumor regions can cycle between hypoxic and normoxic states, a phenomenon known as intermittent or cycling hypoxia, which imposes fluctuating oxidative stress on cancer cells. These oscillations may further exacerbate genomic instability, promote angiogenic bursts, and lead to the selection of more aggressive tumor clones. Targeting hypoxia – whether through normalization of tumor vasculature, inhibition of HIF signaling, or modulation of downstream pathways – remains a key strategy under investigation (Table 1)^[21]^.

**Table 1:** Hypoxia and the lung tumor microenvironment

Aspect	Description	Implications in lung cancer
Definition of hypoxia	Reduced oxygen availability within the tumor microenvironment due to abnormal vasculature and rapid growth.	Promotes metabolic shifts and survival adaptations that support tumor progression.
Hypoxia-inducible factors	Transcription factors (mainly HIF-1α and HIF-2α) stabilized under low oxygen conditions.	Activate genes regulating angiogenesis (VEGF), glycolysis (GLUT1), invasion (MMPs), and survival pathways.
Metabolic reprogramming	Switch from oxidative phosphorylation to anaerobic glycolysis (Warburg effect).	Facilitates energy production despite low oxygen; increases lactate production, promoting acidification and invasion.
Angiogenesis	Formation of new blood vessels driven by hypoxia-induced VEGF expression.	Supports tumor growth and metastasis but often results in dysfunctional vessels, perpetuating hypoxia.
Immune modulation	Hypoxia promotes immunosuppressive microenvironment via recruitment of regulatory T cells and MDSCs.	Leads to immune evasion and reduced efficacy of immunotherapy.
Extracellular matrix (ECM)	Hypoxia influences ECM remodeling by regulating collagen deposition and matrix metalloproteinases.	Enhances tumor invasiveness and facilitates metastatic dissemination.
Therapeutic resistance	Hypoxia reduces sensitivity to radiotherapy and some chemotherapies by altering cell cycle and DNA repair.	Complicates treatment outcomes and contributes to tumor recurrence.

#### Oxidative stress and redox imbalance in lung cancer

Oxidative stress is a central feature of the tumor microenvironment and plays a crucial role in the initiation, progression, and therapeutic resistance of lung cancer. It arises from an imbalance between the production of ROS and the antioxidant defense systems that neutralize them. In lung cancer cells, this imbalance is not merely a by-product of dysregulated metabolism but a tightly regulated process that supports malignant behavior. ROS, which include superoxide anion (O_2_^−^), H_2_O_2_, and hydroxyl radicals (•OH), are generated primarily by mitochondrial respiration, NADPH oxidases (NOX), and other cellular enzymes. At controlled levels, ROS act as signaling molecules to drive proliferation, angiogenesis, and survival; however, excessive accumulation can damage DNA, proteins, and lipids, ultimately leading to cell death^[22]^. Lung cancer cells are uniquely positioned at the interface of environmental oxidants – such as cigarette smoke, air pollutants, and inflammatory stimuli – and intrinsic oncogenic processes that elevate ROS generation. Oncogenes like KRAS and EGFR, frequently mutated in NSCLC, enhance metabolic flux through glycolysis and mitochondrial pathways, thereby increasing ROS production. Meanwhile, tumor suppressor loss, such as that of TP53, impairs the antioxidant response, further tipping the redox balance. Paradoxically, lung cancer cells adapt to these conditions by upregulating antioxidant systems, including glutathione (GSH), thioredoxin (Trx), and superoxide dismutases (SODs), to maintain ROS at pro-tumorigenic but sub-lethal levels^[23]^.

**Figure 1.**
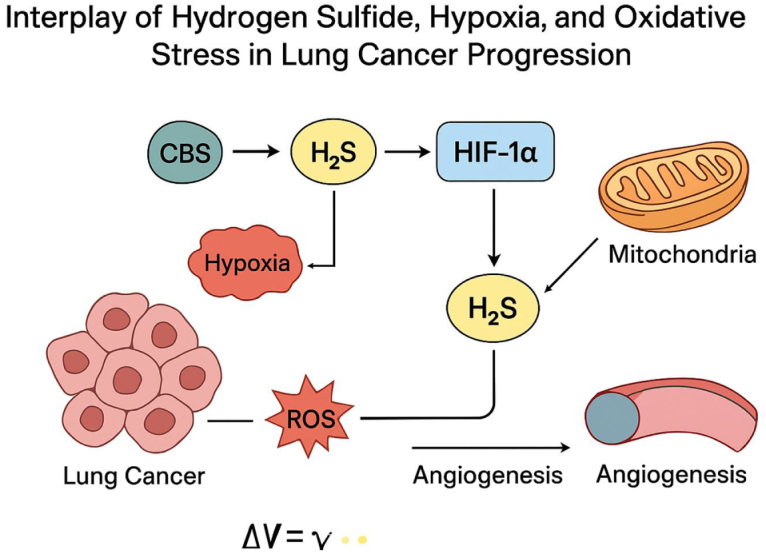
Interplay of hydrogen sulfide, hypoxia, and oxidative stress in lung cancer progression.

This redox adaptation supports several hallmarks of lung cancer. ROS facilitate angiogenesis by activating hypoxia-inducible factors (HIFs) and promoting the expression of VEGF. They also drive EMT, enhance motility and invasion, and modulate matrix metalloproteinase (MMP) activity, contributing to metastasis. Importantly, ROS-induced DNA damage and genomic instability fuel tumor heterogeneity and evolution, complicating therapeutic targeting. Moreover, oxidative stress affects immune responses by altering antigen presentation and dampening cytotoxic immune cell activity, further enabling immune evasion in the tumor microenvironment^[24]^. The relationship between oxidative stress and hypoxia is particularly complex in lung tumors. Hypoxic conditions limit the function of mitochondrial electron transport chains, yet they can paradoxically increase mitochondrial ROS through incomplete oxygen reduction. This coupling of hypoxia and oxidative stress creates a vicious cycle that stabilizes HIF-1α, further promoting tumor adaptation to low oxygen levels. Gasotransmitters like H_2_S and NO also interact with ROS signaling, influencing mitochondrial dynamics, antioxidant enzyme activity, and redox-sensitive transcription factors such as NF-κB and Nrf2. Thus, the oxidative landscape of lung cancer is shaped by a dynamic interplay between metabolic activity, hypoxic signaling, and gasotransmitter modulation (Fig. 1)^[25]^.

Oxidative stress also plays a critical role in therapeutic resistance. Many chemotherapeutic agents, such as cisplatin and paclitaxel, induce cytotoxicity by increasing intracellular ROS to lethal levels. However, cancer cells with enhanced antioxidant capacity can neutralize these effects and survive treatment. Likewise, radiation therapy relies on oxygen-dependent free radical formation to damage DNA; in tumors with high antioxidant buffering or in hypoxic regions where oxygen is limited, radiation is less effective. Targeting redox vulnerabilities – such as by inhibiting glutathione synthesis or thioredoxin reductase – has emerged as a promising strategy to sensitize lung cancer cells to conventional therapies^[26]^. Emerging research also indicates the therapeutic potential of redox-modulating agents. Compounds that either elevate ROS beyond cytotoxic thresholds or disrupt antioxidant systems are being explored as adjuvants to chemotherapy and immunotherapy. Furthermore, precision approaches that exploit synthetic lethality in tumors with defective redox control are under investigation. However, because ROS have dual roles in promoting and inhibiting tumorigenesis, therapeutic interventions must be carefully timed and dosed to avoid unintended consequences, such as promoting cancer stem cell survival or resistance (Table 2)^[27]^.

**Table 2:** Oxidative stress and redox imbalance in lung cancer

Aspect	Description	Implications in lung cancer
Reactive oxygen species (ROS)	Chemically reactive molecules derived from oxygen, including superoxide, hydrogen peroxide, and hydroxyl radicals.	At moderate levels, act as signaling molecules promoting proliferation; excessive ROS cause DNA damage and mutations.
Sources of ROS	Mitochondrial electron transport chain, NADPH oxidases, inflammatory cells, and environmental factors (e.g., smoking).	Contributes to tumor initiation and progression via oxidative DNA damage and chronic inflammation.
Antioxidant defense systems	Enzymes and molecules including superoxide dismutase, catalase, glutathione peroxidase, and glutathione.	Counterbalance ROS to maintain redox homeostasis; dysregulated in lung cancer, leading to oxidative stress.
Redox signaling pathways	ROS modulate pathways such as NF-κB, MAPK, PI3K/AKT, and Nrf2.	Promote survival, inflammation, angiogenesis, and metabolic adaptation in lung tumor cells.
Oxidative DNA damage	ROS induce lesions such as 8-oxoguanine, causing mutations and genomic instability.	Drives oncogenic transformation and tumor heterogeneity.
Mitochondrial dysfunction	Impaired mitochondrial respiration leads to increased ROS production and altered bioenergetics.	Supports metabolic reprogramming and resistance to apoptosis in lung cancer cells.
Therapeutic implications	Redox imbalance affects sensitivity to chemotherapy and radiotherapy.	Targeting redox homeostasis offers potential for overcoming treatment resistance.

#### The triangular crosstalk: H_2_S, hypoxia, and oxidative stress in lung cancer

**Figure 2.**
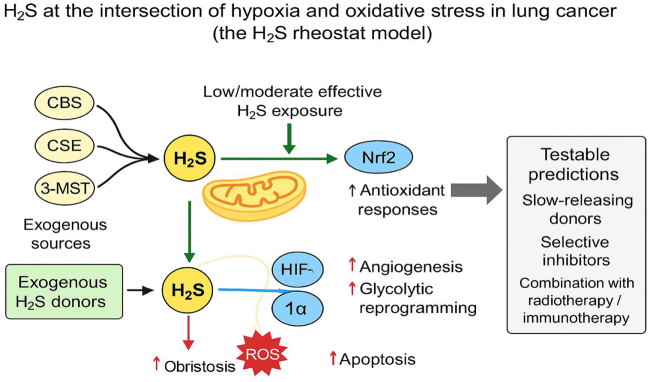
Schematic: hydrogen sulfide (H_2_S) at the intersection of hypoxia and oxidative stress in lung cancer (the H_2_S rheostat model).

The biological interactions among H_2_S, hypoxia, and oxidative stress form a complex and dynamic network that shapes the lung tumor microenvironment. These three elements – each critical in its own right – do not act in isolation. Instead, they engage in a triangular crosstalk that modulates cancer cell behavior, supports tumor adaptability, and influences treatment response^[28]^. H_2_S is a key modulator of redox homeostasis, acting as both an antioxidant and a pro-oxidant depending on its concentration, localization, and the surrounding molecular context. At physiological levels, H_2_S exerts cytoprotective effects by scavenging ROS and enhancing the expression of antioxidant enzymes via activation of Nrf2, a redox-sensitive transcription factor. H_2_S also regulates mitochondrial function by preserving membrane potential, modulating electron transport chain activity, and reducing excessive ROS production. These actions are particularly relevant in the hypoxic regions of lung tumors, where mitochondrial efficiency and redox buffering are vital for cell survival. Under these conditions, H_2_S collaborates with the HIF pathway to promote angiogenesis, metabolic reprogramming, and cellular adaptation (Fig. 2)^[29]^.

Conversely, hypoxia influences the biosynthesis and signaling of H_2_S. Hypoxic stress upregulates the expression of H_2_S-producing enzymes – such as CBS and CSE – through HIF-1α-dependent transcription. This results in increased H_2_S production, particularly within mitochondria, where it supports bioenergetic compensation under oxygen-deficient conditions. In turn, H_2_S stabilizes HIF-1α by inhibiting PHDs, the oxygen-dependent enzymes responsible for HIF degradation. This feedback loop reinforces the hypoxic program, enhancing glycolysis, angiogenesis, and survival signaling. Such synergy between H_2_S and hypoxia contributes to the establishment of a tumor-promoting microenvironment in lung cancer^[30]^. Oxidative stress further complicates this relationship. While ROS can destabilize cellular homeostasis, moderate levels act as signaling intermediates that stimulate H_2_S production through redox-sensitive regulatory mechanisms. In hypoxic lung tumors, where ROS levels can fluctuate due to intermittent oxygenation, H_2_S functions as a redox buffer, limiting oxidative injury and preventing apoptosis. However, chronic oxidative stress may overwhelm the antioxidant capacity conferred by H_2_S, tipping the balance toward DNA damage, mutagenesis, and tumor evolution. Interestingly, high levels of H_2_S can paradoxically enhance ROS production by inhibiting complex IV of the electron transport chain or disrupting mitochondrial integrity, suggesting that the redox role of H_2_S is highly dose- and context dependent^[31]^.

The triangular interaction among H_2_S, hypoxia, and ROS also shapes the immune microenvironment. H_2_S and hypoxia can jointly upregulate immunosuppressive pathways, including PD-L1 expression and regulatory T-cell recruitment, while ROS modulate antigen presentation and dendritic cell function. Together, these elements help lung tumors evade immune surveillance. Furthermore, they contribute to therapeutic resistance: H_2_S enhances antioxidant defenses that counteract chemotherapy-induced oxidative damage, hypoxia limits radiation efficacy, and redox plasticity enables metabolic rewiring that sustains tumor growth under pharmacologic stress^[32]^. From a therapeutic perspective, the crosstalk between H_2_S, hypoxia, and oxidative stress offers multiple intervention points. Inhibiting H_2_S biosynthesis (e.g., via CBS/CSE inhibitors), disrupting HIF signaling, or selectively increasing ROS beyond cytotoxic thresholds are emerging strategies under investigation. Additionally, combining redox-targeting agents with immunotherapy or anti-angiogenic drugs may overcome resistance mechanisms driven by this crosstalk. However, therapeutic targeting of this triad requires precision to avoid triggering compensatory responses that may enhance tumor survival (Table 3)^[33]^.

**Table 3:** The triangular crosstalk: H_2_S, hypoxia, and oxidative stress in lung cancer

Component	Role and mechanism	Impact on lung cancer progression
Hydrogen sulfide (H_2_S)	Produced by CBS, CSE, and 3-MST enzymes; modulates mitochondrial function, angiogenesis, and redox balance.	Promotes tumor growth, metabolic adaptation, and resistance to apoptosis; dual role depending on concentration.
Hypoxia	Stabilizes HIF-1α and HIF-2α under low oxygen; triggers angiogenesis, glycolysis, and survival pathways.	Drives aggressive tumor phenotype, immune suppression, and therapy resistance.
Oxidative stress	Elevated ROS from mitochondrial dysfunction and environmental factors; activates redox-sensitive signaling pathways.	Enhances DNA damage, mutation accumulation, and tumor heterogeneity; can induce apoptosis or survival depending on level.
H_2_S and hypoxia interaction	H_2_S stabilizes HIF-1α and supports hypoxic adaptation by modulating mitochondrial respiration and redox state.	Facilitates cancer cell survival in hypoxic niches and promotes angiogenesis.
H_2_S and oxidative stress interaction	H_2_S acts as an antioxidant by enhancing Nrf2 pathway and glutathione synthesis; at high levels, it can increase ROS.	Protects tumor cells from oxidative damage or induces cell death depending on context.
Hypoxia and oxidative stress interaction	Hypoxia leads to mitochondrial ROS overproduction; ROS further stabilize HIF factors, creating a feedback loop.	Maintains a pro-tumor microenvironment and promotes treatment resistance.

#### Therapeutic implications and targeting H_2_S signaling in lung cancer

The emerging role of H_2_) as a pivotal regulator of lung cancer biology has stimulated interest in the therapeutic potential of targeting H_2_S signaling pathways. As a gasotransmitter, H_2_S participates in numerous tumor-promoting processes, including cell proliferation, angiogenesis, metabolic reprogramming, and resistance to apoptosis – especially under conditions of hypoxia and oxidative stress. Its context-dependent dual role as a cytoprotective and cytotoxic agent provides a unique opportunity for developing precision-based interventions in lung cancer therapy^[34]^. Several studies have highlighted the overexpression of H_2_S-producing enzymes such as CBS, CSE, and 3-MST in lung tumors compared to adjacent normal tissue. Inhibiting these enzymes can reduce intracellular H_2_S levels, leading to impaired mitochondrial bioenergetics, elevated ROS, and apoptosis in cancer cells. For instance, pharmacological inhibition of CBS using aminooxyacetic acid (AOAA) or CSE inhibition with propargylglycine (PAG) has demonstrated anti-tumor effects in preclinical lung cancer models. These inhibitors suppress tumor growth, impair glycolysis, and reduce angiogenic signaling, thereby enhancing the efficacy of chemotherapeutic agents^[35]^.

Moreover, H_2_S-targeting strategies may sensitize lung tumors to oxidative stress-induced cell death. As H_2_S boosts antioxidant defenses via the Nrf2 pathway and maintains mitochondrial redox homeostasis, its inhibition exposes cancer cells to unmitigated oxidative damage. This vulnerability can be exploited in combination with ROS-generating therapies such as chemotherapy, radiotherapy, or redox-cycling agents like arsenic trioxide. Importantly, such combinations may overcome adaptive resistance mechanisms often observed in hypoxic tumor microenvironments, where H_2_S contributes to the stabilization of HIF-1α and suppression of apoptosis^[36]^. Nanotechnology-based delivery systems are also being explored to enhance the therapeutic precision of H_2_S-targeted therapies. For instance, nanocarriers designed to deliver H_2_S inhibitors selectively to hypoxic tumor regions or mitochondria can enhance drug efficacy while minimizing systemic toxicity. Alternatively, “H_2_S donors” – compounds that release H_2_S in a controlled manner – are being studied for their ability to induce ferroptosis or apoptosis in cancer cells when used at higher concentrations. These donor molecules, such as GYY4137 or AP39, show promise for selectively disrupting redox balance and triggering mitochondrial dysfunction in lung cancer cells^[37]^.

Furthermore, H_2_S signaling intersects with several oncogenic and immunomodulatory pathways. It modulates the PI3K/AKT/mTOR axis, influences angiogenesis via VEGF upregulation, and alters immune cell function within the tumor microenvironment. Therapeutically, combining H_2_S inhibition with immune checkpoint inhibitors (e.g., anti-PD-1/PD-L1 therapies) or anti-angiogenic agents may synergize to enhance anti-tumor responses, particularly in patients with hypoxia-driven immune suppression. The immunometabolic regulation mediated by H_2_S also opens the door for integrating metabolic reprogramming strategies into immunotherapy regimens^[36]^. However, challenges remain in translating H_2_S-based therapies into clinical practice. The dual nature of H_2_S as both a tumor promoter and protector of normal physiological processes necessitates precise control of its inhibition or supplementation. Additionally, the spatial heterogeneity of H_2_S levels across tumor regions and the lack of reliable biomarkers for H_2_S activity complicate patient stratification and treatment monitoring. Further research is needed to better define the therapeutic window for H_2_S-targeted interventions, identify predictive biomarkers, and optimize drug delivery platforms^[37]^.

#### Diagnostic and prognostic potential of H_2_S-related biomarkers in lung cancer

H_2_S and its biosynthetic enzymes have emerged not only as functional regulators of lung cancer biology but also as promising diagnostic and prognostic biomarkers. The aberrant expression and activity of H_2_S-generating enzymes – including CBS, CSE, and 3-MST – are frequently observed in various lung cancer subtypes, particularly NSCLC. These alterations are often associated with increased tumor aggressiveness, advanced staging, therapy resistance, and poor clinical outcomes, suggesting that H_2_S-related molecular signatures could serve as valuable tools in clinical oncology^[10]^. Elevated expression of CBS has been reported in lung adenocarcinomas, with strong immunohistochemical staining correlating with tumor size, lymph node metastasis, and vascular invasion. Similarly, upregulation of CSE has been linked to enhanced glycolytic activity and resistance to apoptosis, both hallmarks of malignant transformation. These enzymes can be measured in tumor biopsies, bronchoalveolar lavage fluid, or even circulating tumor-derived exosomes, providing multiple avenues for non-invasive or minimally invasive biomarker development. In particular, CBS expression may serve as a prognostic indicator; studies have shown that patients with high CBS expression in tumor tissue have shorter overall survival and progression-free survival than those with low expression^[38]^.

Beyond the enzymes themselves, the quantification of free or bound H_2_S in biological fluids may offer diagnostic insights. Although technically challenging due to H_2_S’s volatility and rapid metabolism, emerging analytical methods such as high-performance liquid chromatography, mass spectrometry, and fluorescent H_2_S sensors are improving detection accuracy. Elevated circulating H_2_S levels have been observed in some cancer patients and may reflect systemic metabolic alterations driven by tumor burden. Furthermore, H_2_S-related oxidative modifications – such as persulfidation of cysteine residues on redox-sensitive proteins – are being explored as surrogate markers of H_2_S signaling activity, with potential for use in both diagnosis and monitoring of treatment response^[39]^. Integration of H_2_S-related biomarkers with hypoxia and oxidative stress markers could enhance predictive accuracy and clinical relevance. For instance, co-expression of CBS and HIF-1α has been associated with aggressive lung cancer phenotypes and resistance to platinum-based chemotherapy. Similarly, simultaneous assessment of CBS and Nrf2 activity may provide a composite signature of redox adaptability and therapeutic vulnerability. In this context, multimodal biomarker panels incorporating H_2_S enzymes, hypoxic markers (e.g., CAIX, VEGF), and oxidative stress indicators (e.g., 8-OHdG, MDA) may offer superior prognostic value compared to single markers alone^[40–42]^. From a precision medicine standpoint, H_2_S-related biomarkers could also guide therapeutic decision-making. Patients with tumors exhibiting high CBS or CSE expression may be more responsive to therapies targeting H_2_S biosynthesis or redox balance. Conversely, low H_2_S-producing tumors might rely more heavily on alternative metabolic pathways, requiring distinct therapeutic approaches. Furthermore, dynamic monitoring of H_2_S levels during treatment could serve as a pharmacodynamic biomarker, indicating drug efficacy or early resistance^[43–46]^.

### Dualistic role of H_2_S: protective versus tumor-promoting actions

H_2_S exhibits a complex and concentration-dependent duality in cancer biology, functioning as both a cytoprotective and tumor-promoting agent. This paradox arises from its multifaceted influence on redox regulation, mitochondrial activity, and signaling cascades that govern cell proliferation, angiogenesis, and apoptosis. The outcome of H_2_S exposure is dictated by factors such as local concentration, enzymatic source, cellular oxygen tension, and the oxidative environment within the tumor microenvironment^[39,40]^. At physiological or low concentrations, H_2_S acts predominantly as a cytoprotective molecule, preserving redox balance and supporting mitochondrial integrity. It scavenges ROS and reactive nitrogen species, enhancing cellular antioxidant defenses through upregulation of GSH, SOD, and catalase. These actions prevent oxidative DNA damage and apoptosis, thereby maintaining cellular homeostasis. In non-malignant or early-stage cells, such protective mechanisms may help prevent carcinogenesis by mitigating oxidative stress-induced mutations^[41,42]^.

Conversely, at higher concentrations or in tumor settings, H_2_S shifts toward a tumor-promoting phenotype. In lung cancer cells, elevated expression of H_2_S-producing enzymes – particularly CBS and CSE – enhances endogenous H_2_S synthesis. This endogenous overproduction fuels metabolic reprogramming by stimulating glycolytic flux, mitochondrial ATP generation, and biosynthetic pathways essential for rapid tumor proliferation. Moreover, H_2_S supports angiogenesis through stabilization of HIF-1α and subsequent upregulation of VEGF, ensuring sustained oxygen and nutrient delivery to tumor tissues^[43,44]^. Beyond metabolic and angiogenic support, H_2_S modulates cell survival and drug resistance pathways. It activates pro-survival signaling cascades, such as PI3K/Akt and ERK1/2, while inhibiting intrinsic apoptotic mediators including caspase-3 and cytochrome c release. These molecular interactions enhance tolerance to hypoxia and oxidative stress, conditions commonly encountered within the tumor microenvironment, thereby contributing to therapeutic resistance. Experimental inhibition of CBS or CSE in lung cancer cell lines has been shown to suppress tumor growth, elevate ROS accumulation, and sensitize cells to chemotherapeutic agents – highlighting the dependence of malignant cells on H_2_S-driven redox homeostasis^[45]^.

The biphasic or “bell-shaped” dose-response characteristic of H_2_S underscores its duality: submicromolar levels confer cytoprotection and redox stability, whereas micromolar-to-millimolar levels promote tumorigenic adaptations. This dynamic balance resembles a molecular rheostat, where fine-tuning of H_2_S concentrations determines cellular fate under stress conditions. Such complexity presents both a challenge and an opportunity for therapeutic exploitation – where selectively inhibiting tumor-derived H_2_S while preserving physiological signaling in normal tissues could yield novel anticancer strategies^[46]^.

#### Critical appraisal: sources of heterogeneity and conflicting results

Across the studies included in this review, substantial heterogeneity was observed in experimental design, measurement approaches, and biological models, all of which significantly shaped the reported effects of H_2_S in the context of hypoxia and oxidative stress in lung cancer. To systematically account for these differences, we evaluated and annotated each study according to core methodological variables, including the model system used (cell line, primary cells, or animal species), the source of H_2_S (fast- vs. slow-releasing donors, mitochondria-targeted donors, or modulation of endogenous H_2_S-producing enzymes), the nominal concentration or dose range, exposure duration, oxygen level conditions (normoxia vs. precisely defined hypoxia), the specific assays used to quantify reactive oxygen species (ROS) or antioxidant activity, and the primary mechanistic or phenotypic endpoints assessed^[39,40]^. Several important patterns emerged through this structured appraisal. First, donor kinetics emerged as a major determinant of outcome variability. Fast-releasing donors such as NaHS generate sharp, transient spikes in H_2_S concentration and were more commonly associated with cytotoxic or pro-oxidant effects. In contrast, slow-release donors like GYY4137 or mitochondria-targeted formulations such as AP39 generated more sustained, lower-amplitude H_2_S exposure and frequently produced cytoprotective or anti-oxidative signaling effects – even at similar nominal molar concentrations. This underscores that nominal concentration alone is insufficient to interpret H_2_S actions without accounting for release kinetics and chemical behavior in culture media^[41,42]^.

Second, we observed clear concentration-dependent windows of effect. Protective or modulatory effects of H_2_S were typically reported in the low micromolar range, whereas cytotoxicity or oxidative stress induction was described at high micromolar to millimolar concentrations. However, these thresholds varied considerably across cell lines, particularly between lung cancer models and non-malignant bronchial epithelial cells. Such variability suggests that intrinsic differences in metabolic profile, mitochondrial function, and antioxidant capacity strongly influence cellular responsiveness to H_2_S^[43]^. Third, model dependence contributed substantially to divergent results. Cancer cell lines harboring mitochondrial mutations or impaired antioxidant regulation displayed heightened sensitivity to H_2_S modulation, whereas immortalized epithelial cells tended to show more uniform dose-response profiles. Moreover, animal models that genetically manipulate endogenous H_2_S-producing enzymes (CSE or CBS knockdown/knockout) frequently yielded findings that were not fully concordant with pharmacological donor or inhibitor approaches, highlighting the biological complexity of endogenous versus exogenous H_2_S sources^[44]^.

Fourth, the approach used to model hypoxia proved to be another major source of inconsistency. Studies employing rigorously controlled low-oxygen environments (e.g., 1–2% O_2_ in hypoxia chambers) generally observed stronger H_2_S-mediated effects on HIF stabilization and downstream signaling compared with studies relying on chemical hypoxia mimetics. These differences emphasize the importance of oxygen sensing and mitochondrial signaling interfaces that cannot be fully reproduced by chemical methods^[45]^. Finally, heterogeneity was amplified by assay-related limitations, particularly in studies quantifying oxidative stress. A large proportion relied on indirect, artifact-prone methods such as 2′,7′-dichlorofluorescein diacetate (DCFDA) fluorescence. These assays are sensitive to intracellular pH, metal ions, and other variables that can confound interpretations of ROS dynamics. In contrast, studies employing more specific or quantitative techniques – such as electron paramagnetic resonance spectroscopy or targeted redox probes – offered more reliable mechanistic insights^[46,47]^.

#### Future directions and conceptual framework

Despite remarkable progress in understanding the physiological and pathological functions of H_2_S, several critical questions remain unresolved regarding its precise role in lung cancer biology. The emerging evidence portrays H_2_S not as a simple on/off regulator of redox or hypoxic signaling but rather as a context-dependent molecular switch that integrates environmental stress cues to determine cellular survival, proliferation, or death. Future research must therefore move beyond descriptive observations toward mechanistically driven and translationally oriented investigations^[42]^. A key priority lies in quantifying the threshold concentrations at which H_2_S transitions from cytoprotective to tumor-promoting activity. Advanced redox-sensitive probes and high-resolution imaging could help define these dose-response relationships in real time within tumor microenvironments. Equally important is dissecting the spatiotemporal distribution of endogenous H_2_S generated by CBS, CSE, and 3-MST. Mapping enzyme localization in hypoxic tumor cores versus well-oxygenated margins could reveal spatial heterogeneity that influences therapeutic responsiveness^[43]^.

Future studies should also examine interactions between H_2_S and other gasotransmitters, such as NO and CO, which often converge on similar signaling targets. Understanding how these gaseous mediators cooperate or compete under hypoxic stress may uncover novel combinatorial targets for redox modulation. Integrative *omics* approaches – transcriptomics, metabolomics, and proteomics – could delineate downstream networks regulated by H_2_S, providing a systems-level view of its influence on cellular metabolism, angiogenesis, and immune evasion^[44]^. From a translational perspective, selective inhibition of H_2_S-producing enzymes represents a promising therapeutic strategy. The development of highly specific CBS and CSE inhibitors, or targeted delivery systems that restrict H_2_S suppression to tumor tissue, could minimize toxicity to normal cells. Conversely, controlled H_2_S donation through slow-release compounds might enhance cytotoxic ROS accumulation in tumors while preserving physiological signaling elsewhere. These dual approaches – either attenuating or exploiting H_2_S – require careful pharmacodynamic assessment to balance efficacy and safety^[45]^.

Conceptually, the role of H_2_S in lung cancer can be visualized as a redox rheostat positioned between two extremes:

- On one side, low to physiological H_2_S levels sustain antioxidant defense, mitochondrial function, and redox equilibrium.
- On the other, excessive H_2_S production drives metabolic reprogramming, HIF-1α stabilization, and therapy resistance.

The balance between these states is governed by local oxygen tension, enzyme activity, and ROS flux. Targeting this rheostat offers a conceptual framework for future therapeutic design – where fine-tuning H_2_S signaling, rather than complete inhibition, could selectively disrupt tumor adaptation without harming normal tissues. Clinical translation will require biomarker development to identify patients with aberrant H_2_S metabolism. Measuring circulating or tissue H_2_S levels, CBS/CSE expression profiles, or redox signatures could guide patient stratification and therapy optimization. Multicenter studies integrating these biomarkers with pharmacologic interventions would validate the clinical relevance of H_2_S-modulating strategies^[46,47]^.

## Conclusion

H_2_S has emerged as a critical modulator at the intersection of hypoxia and oxidative stress in the lung tumor microenvironment. Its dualistic nature – cytoprotective at physiological levels and cytotoxic at higher concentrations – positions it as both a facilitator of cancer progression and a potential therapeutic target. The interplay between H_2_S signaling, hypoxia-inducible pathways, and redox balance orchestrates a wide range of tumor-promoting processes, including metabolic adaptation, angiogenesis, immune evasion, and resistance to conventional therapies. This triangular crosstalk not only enhances the survival and aggressiveness of lung cancer cells but also contributes to the heterogeneity and complexity of the disease.

Therapeutic strategies aimed at modulating H_2_S levels, inhibiting its biosynthetic enzymes, or disrupting its interaction with hypoxia and oxidative stress signaling have shown promise in preclinical models. Moreover, H_2_S-related enzymes such as CBS, CSE, and 3-MST are gaining recognition as valuable diagnostic and prognostic biomarkers, offering insights into tumor behavior, treatment response, and patient outcomes. The integration of H_2_S biomarkers with existing molecular signatures may enhance the precision of lung cancer diagnosis and support the development of individualized treatment approaches.
